# Green Tea Polyphenols Stimulate Mitochondrial Biogenesis and Improve Renal Function after Chronic Cyclosporin A Treatment in Rats

**DOI:** 10.1371/journal.pone.0065029

**Published:** 2013-06-03

**Authors:** Hasibur Rehman, Yasodha Krishnasamy, Khujista Haque, Ronald G. Thurman, John J. Lemasters, Rick G. Schnellmann, Zhi Zhong

**Affiliations:** 1 Department of Drug Discovery & Biomedical Sciences, Medical University of South Carolina, Charleston, South Carolina, United States of America; 2 Department of Biochemistry & Molecular Biology, Medical University of South Carolina, Charleston, South Carolina, United States of America; 3 Hollings Cancer Center, Medical University of South Carolina, Charleston, South Carolina, United States of America; 4 Department of Pharmacology, University of North Carolina, Chapel Hill, North Carolina, United States of America; 5 Ralph H. Johnson Veterans Affairs Medical Center, Charleston, South Carolina, United States of America; Universidade de Sao Paulo, Brazil

## Abstract

Our previous studies showed that an extract from *Camellia sinenesis* (green tea), which contains several polyphenols, attenuates nephrotoxicity caused by cyclosporine A (CsA). Since polyphenols are stimulators of mitochondrial biogenesis (MB), this study investigated whether stimulation of MB plays a role in green tea polyphenol protection against CsA renal toxicity. Rats were fed a powdered diet containing green tea polyphenolic extract (0.1%) starting 3 days prior to CsA treatment (25 mg/kg, i.g. daily for 3 weeks). CsA alone decreased renal nuclear DNA-encoded oxidative phosphorylation (OXPHOS) protein ATP synthase-β (AS-β) by 42%, mitochondrial DNA (mtDNA)-encoded OXPHOS protein NADH dehydrogenase-3 (ND3) by 87% and their associated mRNAs. Mitochondrial DNA copy number was also decreased by 78% by CsA. Immunohistochemical analysis showed decreased cytochrome *c* oxidase subunit IV (COX-IV), an OXPHOS protein, in tubular cells. Peroxisome proliferator-activated receptor-γ coactivator (PGC)-1α, the master regulator of MB, and mitochondrial transcription factor-A (Tfam), the transcription factor that regulates mtDNA replication and transcription, were 42% and 90% lower, respectively, in the kidneys of CsA-treated than in untreated rats. These results indicate suppression of MB by chronic CsA treatment. Green tea polyphenols alone and following CsA increased AS-β, ND3, COX-IV, mtDNA copy number, PGC-1α mRNA and protein, decreased acetylated PGC-1α, and increased Tfam mRNA and protein. In association with suppressed MB, CsA increased serum creatinine, caused loss of brush border and dilatation of proximal tubules, tubular atrophy, vacuolization, apoptosis, calcification, and increased neutrophil gelatinase-associated lipocalin expression, leukocyte infiltration, and renal fibrosis. Green tea polyphenols markedly attenuated CsA-induced renal injury and improved renal function. Together, these results demonstrate that green tea polyphenols attenuate CsA-induced kidney injury, at least in part, through the stimulation of MB.

## Introduction

Cyclosporin A (CsA) is an important immunosuppressive agent. Even with the development of new immunosuppressants, CsA is still widely used after organ transplantation and for treatment of autoimmune diseases [1]–[3]. Immunosuppressive therapy with CsA is always long-term and results in a number of side effects, the most frequent and severe being nephrotoxicity (e.g. renal dysfunction in up to 30% of patients) [4]–[7].

The mechanisms by which CsA causes nephrotoxicity are not well understood but are thought in part due to calcineurin inhibition [8]. CsA causes acute reversible nephrotoxicity as well as chronic, irreversible nephrotoxicity [7]. Acute CsA renal toxicity is linked to increased renal vascular resistance due to increased vasoconstrictors, decreased vasodilators, activation of renal nerves, and mesangial cell contraction, hypoxia/reperfusion (I/R) and free radical production [5], [7], [9]–[13]. Upregulation of toll-like receptors (TLR) and TNF-α is also involved in CsA nephrotoxicity [14]. Chronic CsA causes decreases of glomerular filtration rates, tubulointerstitial injury, apoptosis, tubular microcalcification, arteriolar hyalinosis, fibrosis, and focal glomerular sclerosis [7]. Mechanisms of CsA chronic damage are less clear compared to the acute nephrotoxicity [7]. Cyclosporine A also upregulates TGF-β expression [15].

Energy supply is essential for cell survival and function. Mitochondrial dysfunction is a common cause of drug/toxicant-induced organ injury and CsA has profound effects on mitochondria. At low concentrations/doses, CsA inhibits the opening of the mitochondrial permeability transition (MPT) pores by binding to cyclophilin D in the matrix and the inner membrane of mitochondria, protecting against I/R injury [16]–[18]. However, at high concentrations/doses, CsA inhibits mitochondrial respiration and decreases ATP production in vivo and in vitro [19]–[21]. It is suspected that poor adaptation to altered mitochondrial energy metabolism is linked to organ vulnerability to CsA toxicity [19]. Mitochondrial biogenesis (MB) is an important adaptation, counteracting mitochondrial dysfunction/toxicity. Calcineurin plays an important role in the expression of peroxisome proliferator-activated receptor-γ coactivator (PGC)-1α [22]–[24], the master regulator of MB. Whereas CsA is a potent calcineurin inhibitor. it is possible that CsA suppresses MB to induce nephrotoxicity.

Green tea polyphenols are free radical and singlet oxygen scavengers. Beneficial effects of green tea polyphenols in the prevention/treatment of cardiovascular, hepatic, renal, neural, pulmonary and intestinal diseases, cancer, diabetes, arthritis, shock, and decreases in ischemia/reperfusion injury and drug/chemical toxicity in various organs/tissues have been widely reported and many of these effects are presumably due to their antioxidant and anti-inflammatory properties [25]–[40]. Our previous study showed that a *Camellia sinenesis* (green tea) extract, which contains high levels of plant polyphenols (e.g. epigallocatechin gallate, epigallocatechin, epicatechin, and catechin), attenuated CsA nephrotoxicity, in part, by scavenging free radicals [31]. Recent studies showed that isoflavones are effective MB stimulators and improve mitochondrial function after renal I/R injury, diabetes, chronic heart failure, and aging [41]–[45]. Stimulation of MB increases mitochondrial proteins and mass, improves function, and is an attractive strategy for promoting cell repair and regeneration, preserving organ function and treating a number of pathologies resulting from damage/inhibition of mitochondrial function [42]–[50]. Thus, this study was designed to explore the effects of green tea polyphenols on renal MB after chronic CsA treatment.

## Materials and Methods

### Cyclosporin A and Polyphenol Treatments

CsA (Sandimmune oral solution) was obtained from Novartis (Basel, Switzerland). Green tea extracts, produced by Taiyo Kagaku Co. (Yokkaichi, Mie, Japan), contained 85% polyphenols by weight. Components of polyphenols in the extract was determined by high performance liquid chromatography (HPLC) as described previously [31] and included epigallocatechin gallate (47.2% of total polyphenols), epigallocatechin (11.0%), gallocatechin gallate (11.0%), epicatechin gallate (10.8%), gallocatechin (8.6%), epicatechin (8.4%), and catechin (3.0%).

Male Sprague-Dawley rats (200–250 g) were fed a semi-synthetic powdered diet (AIN 76, Dyets Inc. Bethlehem, PA) containing 0% or 0.1% green tea polyphenolic extract starting 3 days prior to and lasting throughout CsA or vehicle treatment. Previous studies showed that 0.1% green tea extract in the diet blunted CsA renal toxicity, hepatic I/R injury, cholestatic liver fibrosis and inhibited development of renal cell tumors in rats [29], [31], [40], [51]. Daily food consumption was not different between the groups with or without green tea polyphenolic extract addition. Average polyphenolic extract intake calculated by the daily food consumption and polyphenolic content in the diet was ∼80 mg/kg/day. CsA oral solution or its vehicle (Cremophor EL 20 mg/ml in 12.5% dehydrated alcohol) was further diluted in olive oil. Previous studies have shown that higher doses of CsA are required in rats to cause renal damages that are similar to the damages observed in humans [6], [11], [12]. Therefore, CsA (25 mg/kg, 0.25 mL/100 g body weight) or an equivalent volume of vehicles was gavaged daily for 21 days.

### Serum Creatinine, Renal Histology and Immunohistological Staining

At 21 days after CsA treatment, rats were anesthetized with pentobarbital (50 mg/kg, *i.p*), and blood was collected from the vena cava. Serum creatinine was determined using a kit from Sigma-Aldrich Co. (St. Louis, MO). The left kidney was rinsed with 5 ml normal saline, perfusion-fixed with 10% formaldehyde in phosphate buffered saline, then removed and placed in the same fixative for 48 h. Sections were stained with hematoxylin-eosin (H&E) and analyzed microscopically for pathological changes. Apoptosis was assessed by terminal deoxynucleotidyl transferase dUTP nick-end labeling (TUNEL) using an in situ cell death detection kit from Roche Diagnostics (Penzberg, Germany) [52]. Tubules in the cortex with and without injury (vacuolization, loss of brush border, dilatation, necrosis, atrophy, and calcification), leukocytes, and TUNEL-positive cells were counted in a blinded manner in 10 randomly selected fields per slide under a Nikon Optiphot-2 microscope (Nikon Instruments Inc., Melville, NY) using a 40x objective lens after H&E and TUNEL staining, respectively. Renal fibrosis was detected using the Masson’s Trichrome staining. Immunohistological staining of cytochrome *c* oxidase subunit IV (COX-IV), a nuclear DNA (nDNA)-encoded mitochondrial oxidative phosphorylation (OXPHOS) protein, was performed as described elsewhere [53] using a specific antibody against COX-IV at a dilution of 1∶200.

### Detection of Mitochondrial DNA (mtDNA) Copy Number, ATP Synthase-β (AS-β), NADH Dehydrogenase-3 (ND3), PGC-1α, and Mitochondrial Transcription Factor A (Tfam) mRNAs by Quantitative Real-time PCR (qPCR)

Relative quantities of mtDNA content in the kidney were determined using qPCR [46]. Total genomic DNA was extracted using a DNeasy Blood and Tissue kit (Qiagen, Valencia, CA). Mitochondrial DNA copy number was assessed by quantification of mtDNA-encoded NADH dehydrogenase-1 (ND1) gene using a CFX96 Real Time-PCR Detection System (Bio-Rad, Hercules, CA) and normalized against the nuclear-encoded POU class 5 homeobox 1 (Pou5f1) gene. Primer sequences used are listed in Table 1.

**Table 1:** Real-Time PCR Primers.

*DNAs/mRNAs*		*Primers*
**AS-β**	Forward:	5′- TTG CTG AGG TCT TCA CAG GTC ACA-3′
	Reverse:	5′- CAG CCT TTG CCA CAG CTT CTT CAA-3′
**ND1**	Forward:	5′- TTA ATT GCC ATG GCC TTC CTC ACC-3′
	Reverse:	5′- TGG TTA GAG GGC GTA TGG GTT CTT-3′
**ND3**	Forward:	5′- CAA CAA GTT CTG CAC GCC TTC CTT-3′
	Reverse:	5′- TTG TTT GAA TCG CTC ATG GGA GGG-3′
**Tfam**	Forward:	5′- GAT GAG TCA CCT CAA GGG AAA TTG-3′
	Reverse:	5′- GTC ATC TAG TAA AGC CCG GAA GGT-3′
**Pou5f1**	Forward:	5′- AGG TGT TCA GCC AGA CAA CCA TCT-3′
	Reverse:	5′- TCT CGT TGT TGT CAG CTT CCT CCA-3′
**HPRT**	Forward	5′- TCG AAG TGT TGG ATA CAG GCC AGA-3′
	Reverse:	5′-TAC TGG CCA CAT CAA CAG GAC TCT-3′

AS-β, ATP synthase-β; ND1, NADH dehydrogenase-1; ND3, NADH dehydrogenase-3; Tfam, mitochondrial transcription factor-A; Pou5f1, POU class 5 homeobox 1; HPRT, hypoxanthine phospho-ribosyl-transferase.

Quantitative real-time PCR of mRNAs was performed as described elsewhere [54]. After total RNA isolation from kidney tissue with Trizol (Invitrogen, Grand Island, NY), single stranded cDNAs were synthesized from RNA (2 µg) using a Bio-Rad iScript cDNA Synthesis kit (Bio-Rad, Hercules, CA). Quantitative real-time PCR was conducted using the primer sequences in Table 1. The abundance of mRNAs was normalized against hypoxanthine phospho-ribosyl-transferase (HPRT) using the ΔΔ*Ct* method.

### Immunoprecipitation of PGC-1α

Immunoprecipitation was performed as described elsewhere [55]. Kidneys were homogenized and extracted in ice-cold lysing buffer. Immunoprecipitations were carried out with protein lysate (500 µg protein as determined by the Bradford assay) and PGC-1α antibody (5 µg) using a Catch and Release v2.0 Reversible Immunoprecipitation System (Millipore, Billerica, MA). Protein content in the immunoprecipitates was determined by the Bradford assay. Acetylated lysine residues and PGC-1α were determined by immunoblotting [54] using corresponding specific antibodies (Cell Signaling Technology, Danvers, MA and Santa Cruz Biotech., Santa Cruz, CA, respectively).

### Immunoblotting

Proteins in renal tissue extracts were detected by immunoblot analysis as previously described [54] using primary antibodies specific for AS-β, neutrophil gelatinase-associated lipocalin (NGAL), Tfam (GenWay Biotech, San Diego, CA), cleaved caspase-3 (Cell Signaling Technology, Danvers, MA), ND3 and PGC-1α (Santa Cruz Biotech., Santa Cruz, CA) at concentrations of 1∶100 to 1000, and actin (ICN, Costa Mesa, CA) at a concentration of 1∶3000 at 4°C over night, respectively. Horseradish peroxidase-conjugated secondary antibodies were applied afterwards, and detection was by chemiluminescence (Pierce Biotec., Rockford, IL).

### Statistical Analysis

Groups were compared using ANOVA plus a Student-Newman-Keuls posthoc test. There were 4 rats per group for all parameters. Data shown are means±S.E.M. Differences were considered significant at p<0.05.

### Ethics Statement

All animals were given humane care in compliance with institutional guidelines using protocols approved by the Institutional Animal Care and Use Committee of the University of North Carolina. All surgery was performed under sodium pentobarbital anesthesia (50 mg/kg, i.p.).

## Results

### CsA Decreases mtDNA Copy Number in the Kidney: Reversal by Green Tea Polyphenols

**Figure 1:**
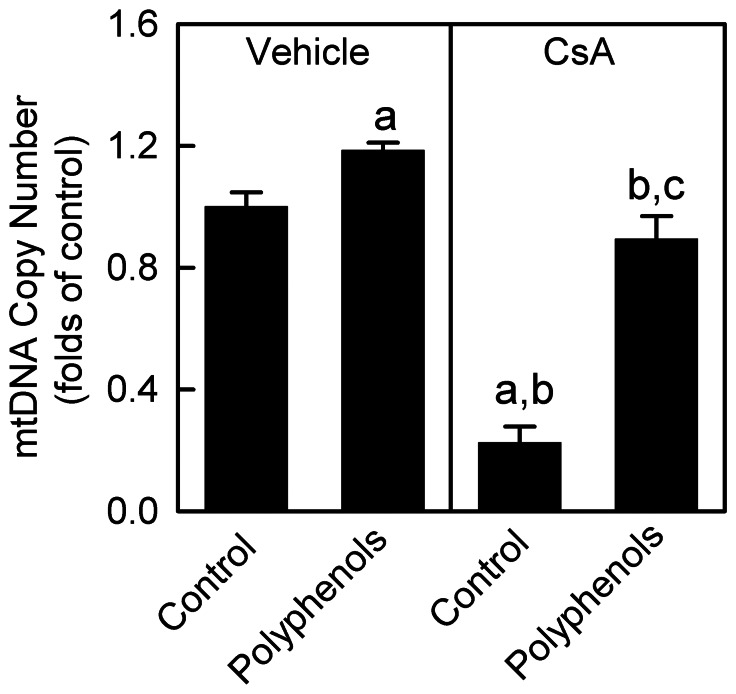
Decreases of mitochondrial DNA copy numbers by cyclosporin A (CsA): reversal by green tea polyphenols. Rats were fed semi-synthetic powdered diets containing 0% (**control**) or 0.1% polyphenol extracts (**polyphenols**) starting 3 days prior to CsA treatment (25 mg/kg, *i.g.* for 3 weeks). Renal mitochondrial DNA copy numbers were determined by qPCR. a, p<0.05 *vs.* control diet+vehicle; b, p<0.05 *vs.* polyphenol-containing diet+vehicle; c, p<0.05 *vs.* control diet+CsA (n = 4 per group).

CsA treatment causes changes in high-energy phosphate homeostasis in tissues [19]–[21]. Mitochondrial DNA is responsible for synthesis of crucial mitochondrial OXPHOS proteins, and proper function of mitochondrial respiration requires an adequate copy number of mtDNA per cell [49], [56]. Therefore, we examined the alterations in mtDNA in the kidney after vehicle and CsA treatment. Renal mtDNA copy number decreased by 78% after chronic CsA treatment (Fig. 1). Polyphenols increased mtDNA copy number by 19% in rats treated with the vehicle and recovered mtDNA copy number to ∼90% of control levels after chronic CsA treatment (Fig. 1).

### CsA Decreases Mitochondrial OXPHOS Proteins in the Kidney: Reversal by Green Tea Polyphenols

**Figure 2:**
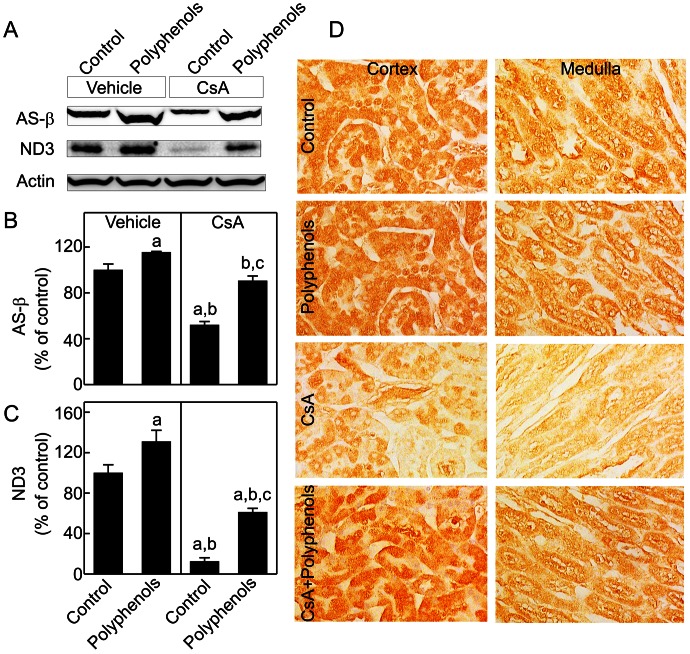
Decreases of mitochondrial oxidative phosphorylation proteins by CsA: reversal by green tea polyphenols. Rats were fed semi-synthetic powdered diets containing 0% (**Control**) or 0.1% polyphenol extracts (**Polyphenols**) starting 3 days prior to CsA treatment (25 mg/kg, *i.g.* for 3 weeks). Renal mitochondrial oxidative phosphorylation proteins ATP synthase-β (AS-β, 55 kDa) and NADH dehydrogenase-3 (ND3, 24 kDa) were determined by immunoblotting and representative images are shown in **A**. AS-β (**B**) and ND3 (**C**) were quantified by densitometry. a, p<0.05 *vs.* control diet+vehicle; b, p<0.05 *vs.* polyphenol-containing diet+vehicle; c, p<0.05 *vs.* control diet+CsA (n = 4 per group). Cytochrome *c* oxidase subunit IV was detected by immunohistochemistry (**D**). Representative images of 4 rats per group are shown.

The majority of mitochondrial proteins are encoded by nuclear DNA (nDNA) [56], [57]. We examined a subunit of F_0_F_1_ATPase, AS-β, that is encoded by nDNA after chronic CsA treatment. CsA decreased AS-β by 48% (Fig. 2A,B). Polyphenols increased AS-β 15% above control levels in the kidneys from vehicle-treated rats and recovered AS-β to ∼91% of control levels in the kidneys from CsA-treated rats. ND3, a mtDNA-encoded mitochondrial OXPHOS protein, decreased 88% after chronic CsA treatment (Fig. 2A,C). Polyphenols increased ND3 to 31% above control levels in the kidneys of vehicle-treated rats and recovered ND3 to 61% of control levels in the kidneys of CsA-treated rats (Fig. 2A,C).

Expression of COX-IV, a nDNA-encoded mitochondrial OXPHOS protein, was observed in various renal cells by immunohistochemical staining (Fig. 2D). In control rats, immunoreactivity of COX-IV was localized throughout the tubular cells in kidney cortex and the medulla, particularly in the proximal tubules. COX-IV staining in tubular cells was punctate, consistent with mitochondrial localization. In contrast, immunoreactivity of COX-IV was low in glomeruli (data not shown). After CsA treatment, COX-IV immunoreactivity decreased substantially in tubular cells in the cortex and medulla. Polyphenols increased COX-IV immunoreactivity in tubular cells after CsA treatment.

### CsA Decreases AS-β and ND3 mRNAs in the Kidney: Recovery by Green Tea Polyphenols

**Figure 3:**
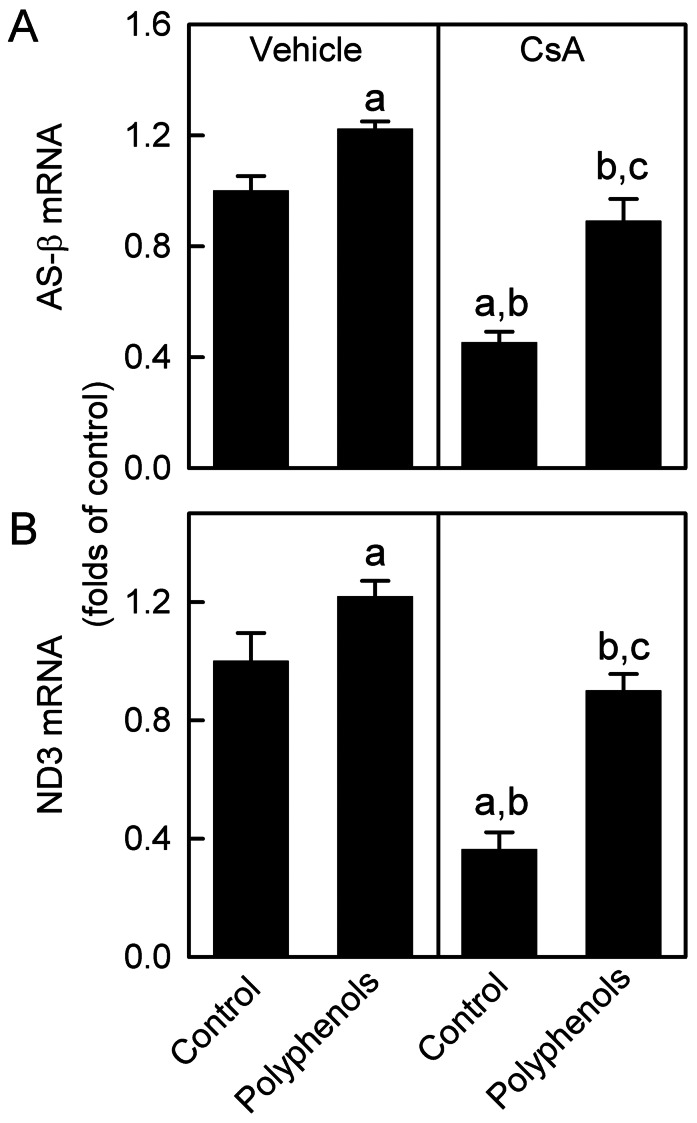
Decreases of ATP synthase-β and NADH dehydrogenase-3 mRNAs by CsA: reversal by green tea polyphenols. Rats were fed semi-synthetic powdered diets containing 0% (**Control**) or 0.1% polyphenol extracts (**Polyphenols**) starting 3 days prior to CsA treatment (25 mg/kg, *i.g.* for 3 weeks). Renal ATP synthase-β (**AS-β**) and NADH dehydrogenase-3 (**ND3**) mRNAs determined by qPCR are shown in **A** and **B**, respectively. a, p<0.05 *vs.* control diet+vehicle; b, p<0.05 *vs.* polyphenol-containing diet+vehicle; c, p<0.05 *vs.* control diet+CsA (n = 4 per group).

Since AS-β and ND3 were decreased by CsA, we investigated the mRNA levels of these proteins. AS-β mRNA decreased 55% by CsA treatment (Fig. 3A). Polyphenols increased AS-β mRNA 22% above control levels in the kidneys of vehicle-treated rats and recovered AS-β mRNA to 89% of control values in the kidneys of CsA-treated rats. Renal ND3 mRNA was 64% lower in CsA-treated rats compared to vehicle-treated rats (Fig. 3B). ND3 mRNA increased 21% in vehicle-treated rats and recovered to ∼90% of control values in CsA-treated rats. Together, we suggest that decreases in AS-β and ND3 proteins are due, at least in part, to suppression of their mRNAs and these effects were reversed by green tea polyphenols.

### Green Tea Polyphenols Increase PGC-1α mRNA and Activation after CsA Treatment

**Figure 4:**
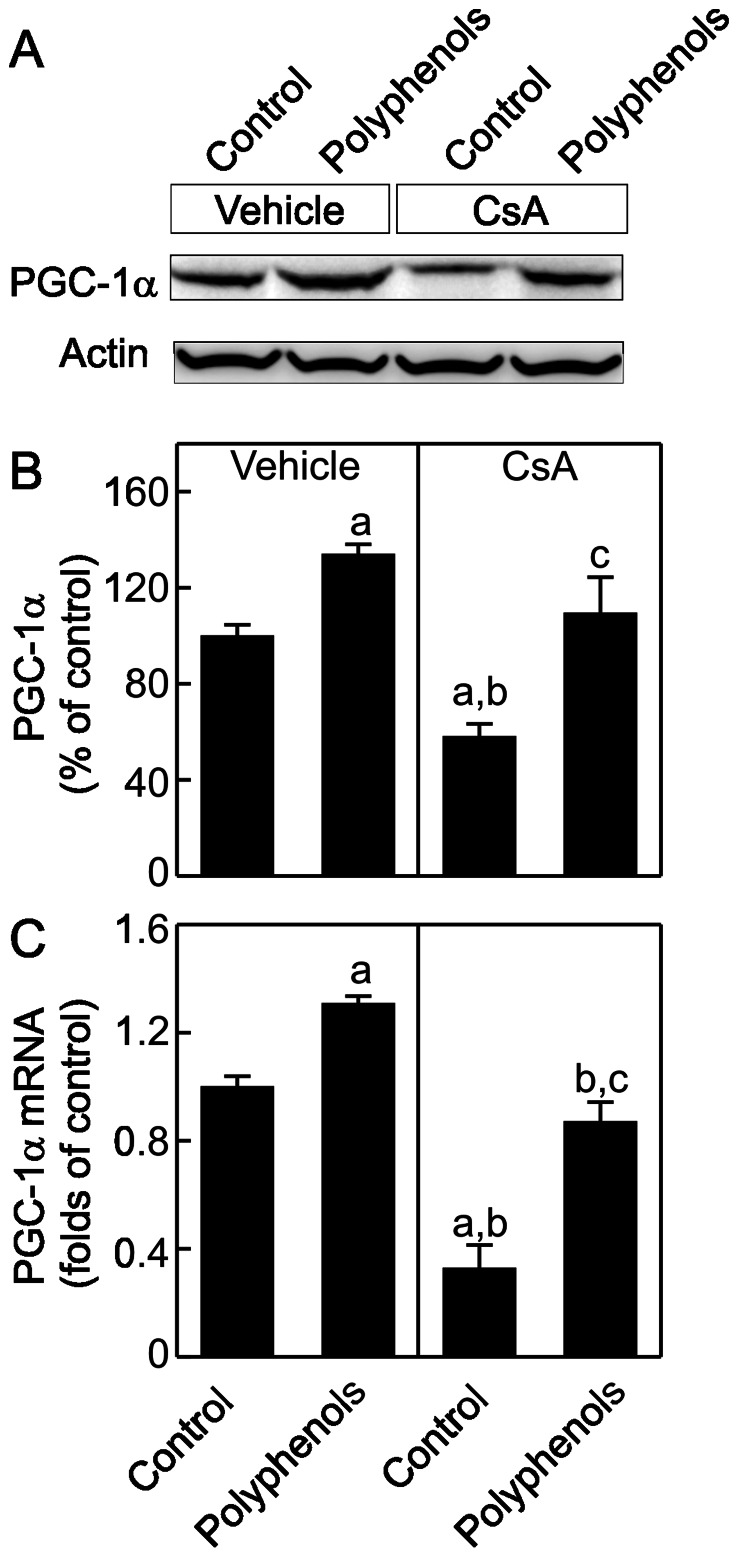
Suppression of peroxisome-proliferator-activated receptor gamma coactivator-1α (PGC-1α) expression by CsA: reversal by green tea polyphenols. Rats were fed semi-synthetic powdered diets containing 0% (**Control**) or 0.1% polyphenol extracts (**Polyphenols**) starting 3 days prior to CsA treatment (25 mg/kg, *i.g.* for 3 weeks). Renal PGC-1α (90 kDa) and actin (43 kDa) were determined by immunoblotting. Representative images are shown in **A** and quantification by densitometry is shown in **B**. PGC-1α mRNA was determined by qPCR (**C**). a, p<0.05 *vs.* control diet+vehicle; b, p<0.05 *vs.* polyphenol-containing diet+vehicle; c, p<0.05 *vs.* control diet+CsA (n = 4 per group).

PGC-1α plays a key role in the control of MB and mtDNA maintenance [58]. We investigated whether CsA alters PGC-1α levels in the kidney. PGC-1α was 42% lower in the kidneys of CsA-treated than vehicle-treated rats (Fig. 4A and B). Polyphenols increased PGC-1α by 34% in the kidneys of vehicle-treated rats and increased PGC-1α to control levels in the kidneys of CsA-treated rats. We further examined whether polyphenols increased PGC-1α mRNA. CsA decreased PGC-1α mRNA by 67% (Fig. 4C). Polyphenols slightly increased PGC-1α mRNA in the kidneys of vehicle-treated rats and recovered PGC-1α mRNA in the kidneys of CsA-treated rats to 87% of control levels.

**Figure 5:**
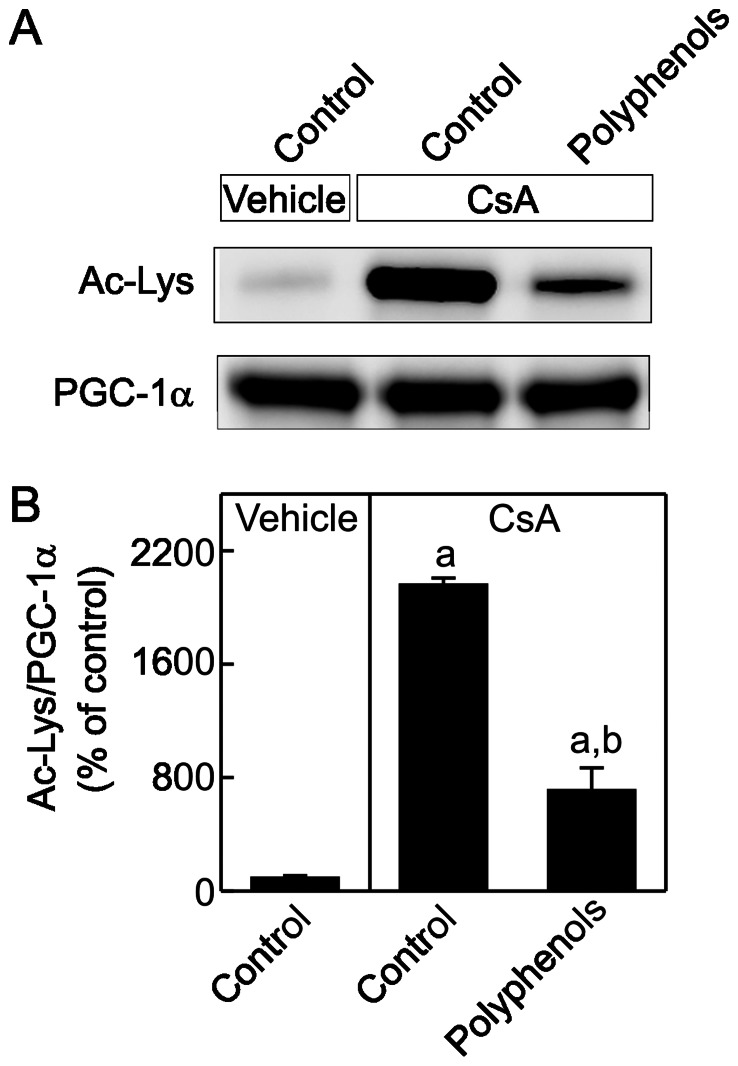
Suppression of peroxisome proliferator-activated receptor gamma coactivator-1α (PGC-1α) activation by CsA: reversal by green tea polyphenols. Rats were fed semi-synthetic powdered diets containing 0% (**Control**) or 0.1% polyphenol extracts (**Polyphenols**) starting 3 days prior to CsA treatment (25 mg/kg, *i.g.* for 3 weeks). Renal PGC-1α was immunoprecipitated, equally loaded, and acetylated lysine (Ac-Lys) and PGC-1α were determined by immunoblotting. Representative images are shown in **A** and quantification by densitometry is shown in **B**. a, p<0.05 *vs.* control diet+vehicle; b, p<0.05 *vs.* polyphenol-containing diet+vehicle; c, p<0.05 *vs.* control diet+CsA (n = 4 per group).

PGC-1α activity is higher after de-acetylation [46]. PGC-1α was immunoprecipitated and acetylated lysine residues were detected by immunoblotting (Fig. 5A and B). Acetylation of PGC-1α was increased substantially after chronic CsA treatment and polyphenols decreased acetylation of PGC-1α, indicating enhanced PGC-1α activation.

### Green Tea Polyphenols Increase Tfam Synthesis after CsA Treatment

**Figure 6:**
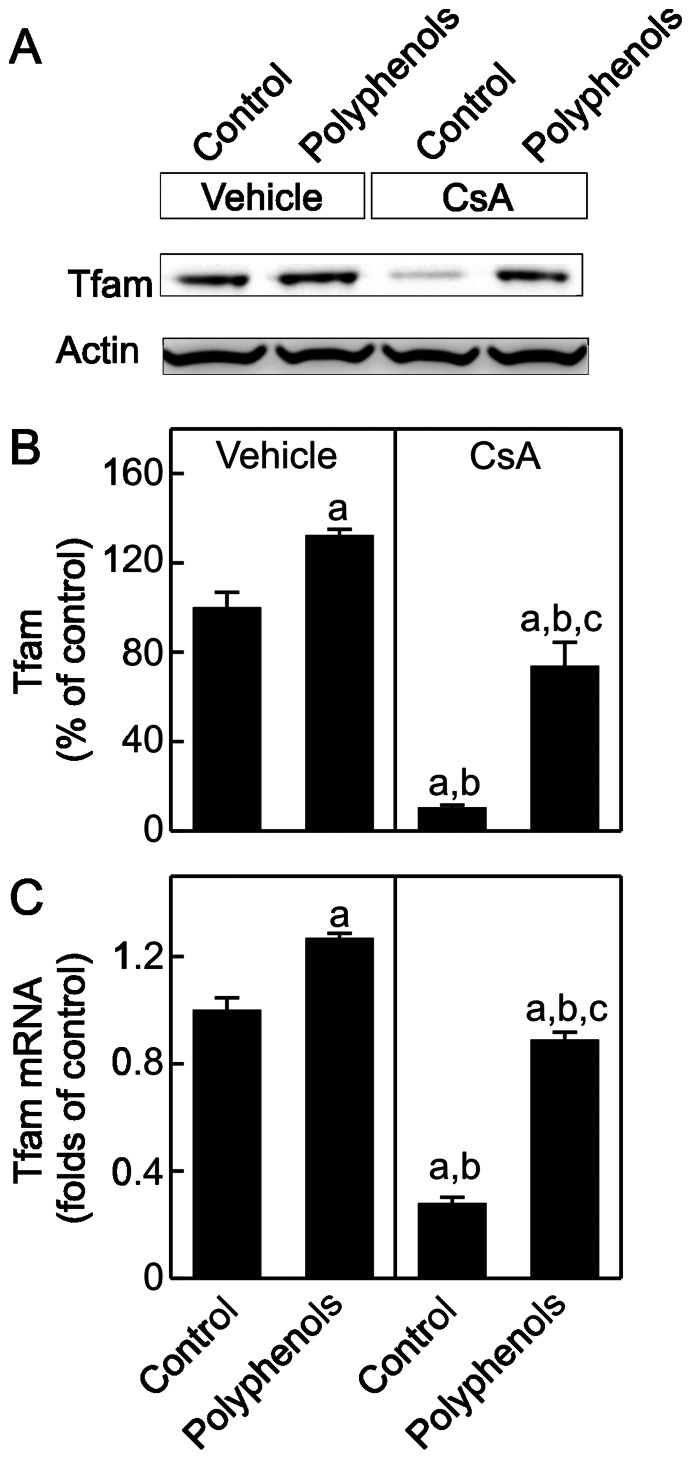
Suppression of mitochondrial transcription factor A (Tfam) expression by CsA: reversal by green tea polyphenols. Rats were fed semi-synthetic powdered diets containing 0% (**Control**) or 0.1% polyphenol extracts (**Polyphenols**) starting 3 days prior to CsA treatment (25 mg/kg, *i.g.* for 3 weeks). Renal Tfam protein (30 kDa) was determined by immunoblotting. Representative images are shown in **A** and quantification by densitometry is shown in **B**. Tfam mRNA was determined by qPCR (**C**). a, p<0.05 *vs.* control diet+vehicle; b, p<0.05 *vs.* polyphenol-containing diet+vehicle; c, p<0.05 *vs.* control diet+CsA (n = 4 per group).

Tfam is a transcription factor that regulates the replication and transcription of the mitochondrial genome, thus playing a critical role in controlling MB [59]. Tfam decreased by almost 90% after CsA treatment (Fig. 6A and B), consistent with decreased mtDNA copy number and mtDNA-encoded ND3 transcription. Expression of Tfam is controlled by PGC-1α [59]. Consistent with decreased PGC-1α expression and activity, Tfam mRNA also decreased after CsA treatment (Fig. 6C), indicating suppressed Tfam transcription. Polyphenols elevated renal Tfam mRNA and protein modestly in vehicle-treated rats and largely reversed the decreases of renal Tfam mRNA and protein in CsA-treated rats.

### Green Tea Polyphenols Attenuate Kidney Injury and Improve Kidney Function after CsA Treatment

**Figure 7:**
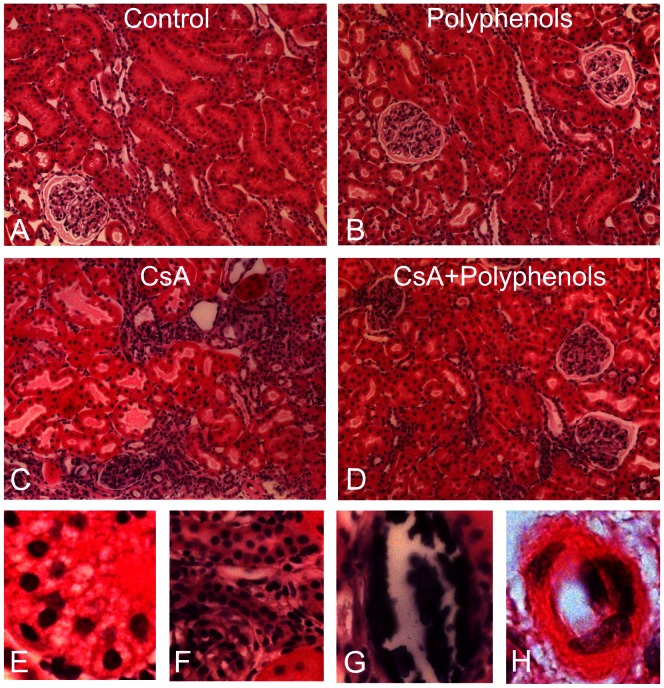
Dietary polyphenols minimize CsA-induced pathological changes in the kidney. Rats were fed semi-synthetic powdered diets containing 0% (**Control**) or 0.1% polyphenol extracts (**Polyphenols**) starting 3 days prior to CsA treatment (25 mg/kg, *i.g.* for 3 weeks). Representative images of H&E-stained kidney sections are shown. **A**, control diet plus vehicle; **B**, 0.1% polyphenols plus vehicle; **C, E, F, G, and H**, control diet plus CsA; **D**, polyphenols plus CsA. **E**, tubular cell vacuolization; **F**, tubular atrophy; **G**, calcification; **H**, arteriolar hyalinosis.

**Figure 8:**
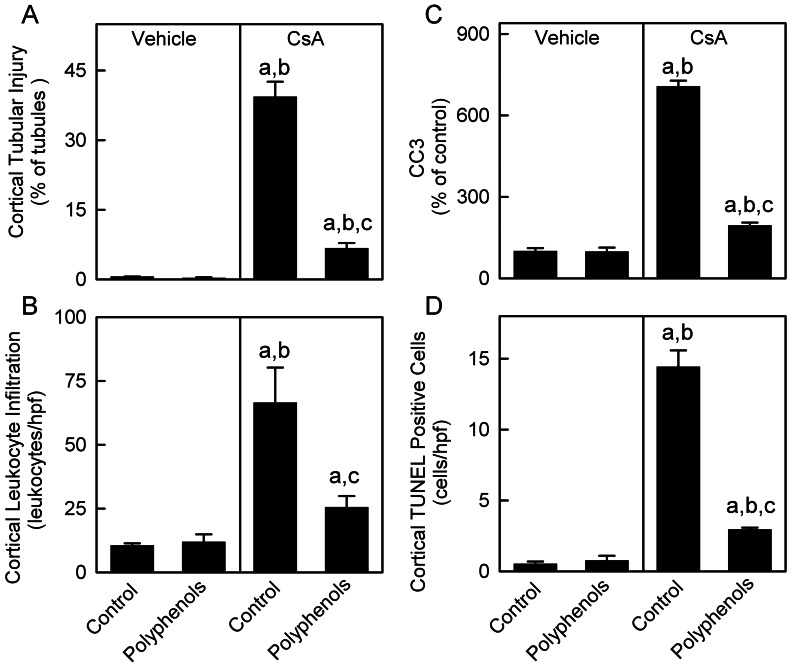
Dietary polyphenols minimize CsA-induced tubular injury and leukocyte infiltration in the cortex. Rats were fed semi-synthetic powdered diets containing 0% (**Control**) or 0.1% polyphenol extracts (**Polyphenols**) starting 3 days prior to CsA treatment (25 mg/kg, *i.g.* for 3 weeks). Tubules with and without injury, leukocytes, and TUNEL-positive cells in the cortex were counted in a blinded manner in 10 randomly selected fields per slide using a 40x objective lens after H&E and TUNEL staining, respectively. Percentages of tubules with injury are shown in **A.** The average numbers of leukocytes and TUNEL-positive cells per high power field (hpf) are shown in **B** and **D**, respectively. Cleaved caspase-3 (CC3) in kidney tissue was detected by immunoblotting (17 kDa) and quantified by densitometry (**C)**. a, p<0.05 *vs.* control diet+vehicle; b, p<0.05 *vs.* polyphenol-containing diet+vehicle; c, p<0.05 *vs.* control diet+CsA (n = 4 per group).

Renal histology was examined after treatment with CsA for 3 weeks (Fig. 7). The kidneys of rats on the control and polyphenol diets that received vehicle treatment exhibited normal histology (A and B). CsA treatment caused a loss of brush border and dilatation of proximal tubules (C), tubular atrophy (C and F), vacuolization (E), calcification (G), cast formation, arteriolar hyalinosis (H) and leukocyte infiltration, most overtly in the cortex, consistent with previous reports [31], [60]. Pathological changes occurred in 39% of tubules in the cortex and leukocytes increased from 10/high power field (hpf) to 66/hpf after CsA treatment (Fig. 8A,B). Polyphenols decreased tubular injury after CsA treatment to 7% and leukocyte to 25/hpf (Fig. 8 A,B).

Cleaved caspase-3 was barely detectable in the kidneys from vehicle-treated rats with or without polyphenol treatment. CsA increased cleaved caspase about 7-fold (Fig. 8C) and this effect was largely blunted by polyphenols. TUNEL-positive cells in the cortex were 0.5–0.8/hpf in vehicle-treated rats with or without polyphenol treatment. TUNEL-positive cells increased to ∼14/hpf after CsA treatment in the absence of polyphenols and was decreased to 3/hpf in the presence of polyphenols (Fig. 8D). These data show that CsA caused apoptosis and this effect was blunted by polyphenols.

**Figure 9:**
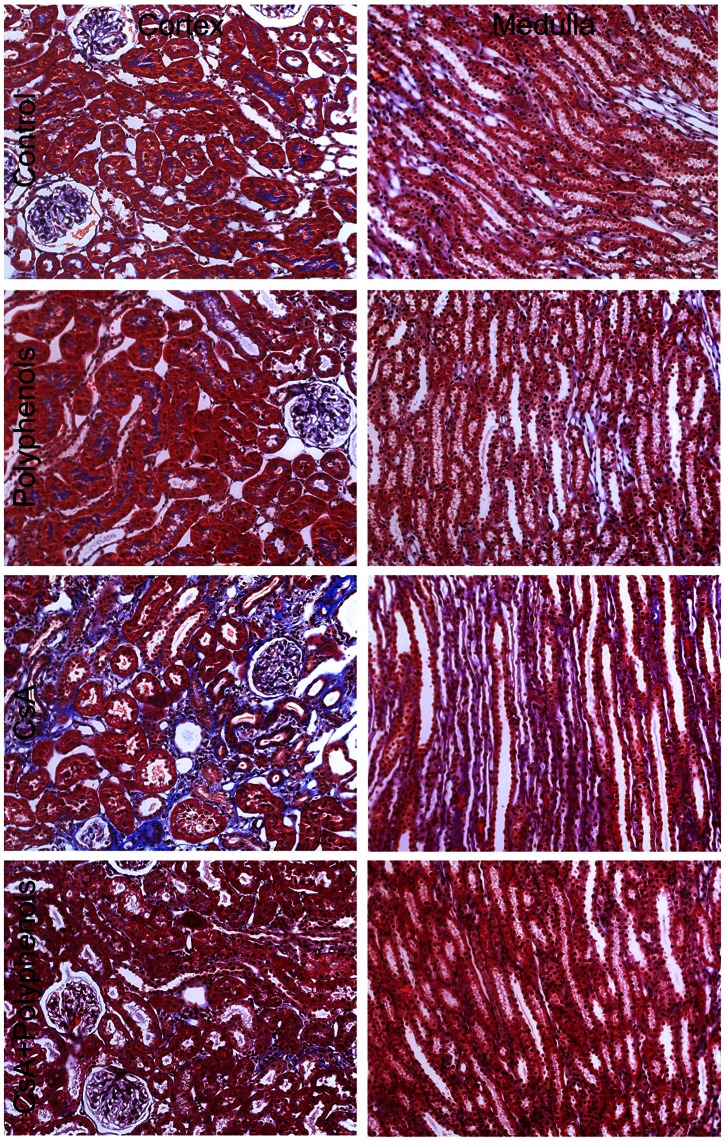
Dietary polyphenols attenuate CsA-induced renal fibrosis. Rats were fed semi-synthetic powdered diets containing 0% (**Control**) or 0.1% polyphenol extracts (**Polyphenols**) starting 3 days prior to CsA treatment (25 mg/kg, *i.g.* for 3 weeks). Representative images of Trichrome-stained kidney sections are shown. **1st row**, control diet plus vehicle; **2nd row**, 0.1% polyphenols plus vehicle; **3rd row**, control diet plus CsA; **4th row**, polyphenols plus CsA (n = 4 per group).

Renal fibrosis was revealed using Masson’s Trichrome staining (Fig. 9). In the kidneys from vehicle-treated rats with or without polyphenol treatment, Trichrome staining was rare in the interstitium. A small amount of blue Trichrome staining appeared in the brush borders, perhaps reflecting the microfilaments in the microvilli of tubular cells. After CsA treatment, blue staining in the brush borders of tubular cells disappeared and was replaced with wide-spread interstitial fibrosis, most overtly in the cortex but also observable in the medulla. Thickening of Bowman’s capsule also occurred in some glomeruli. Renal fibrosis was blunted markedly by polyphenols.

**Figure 10:**
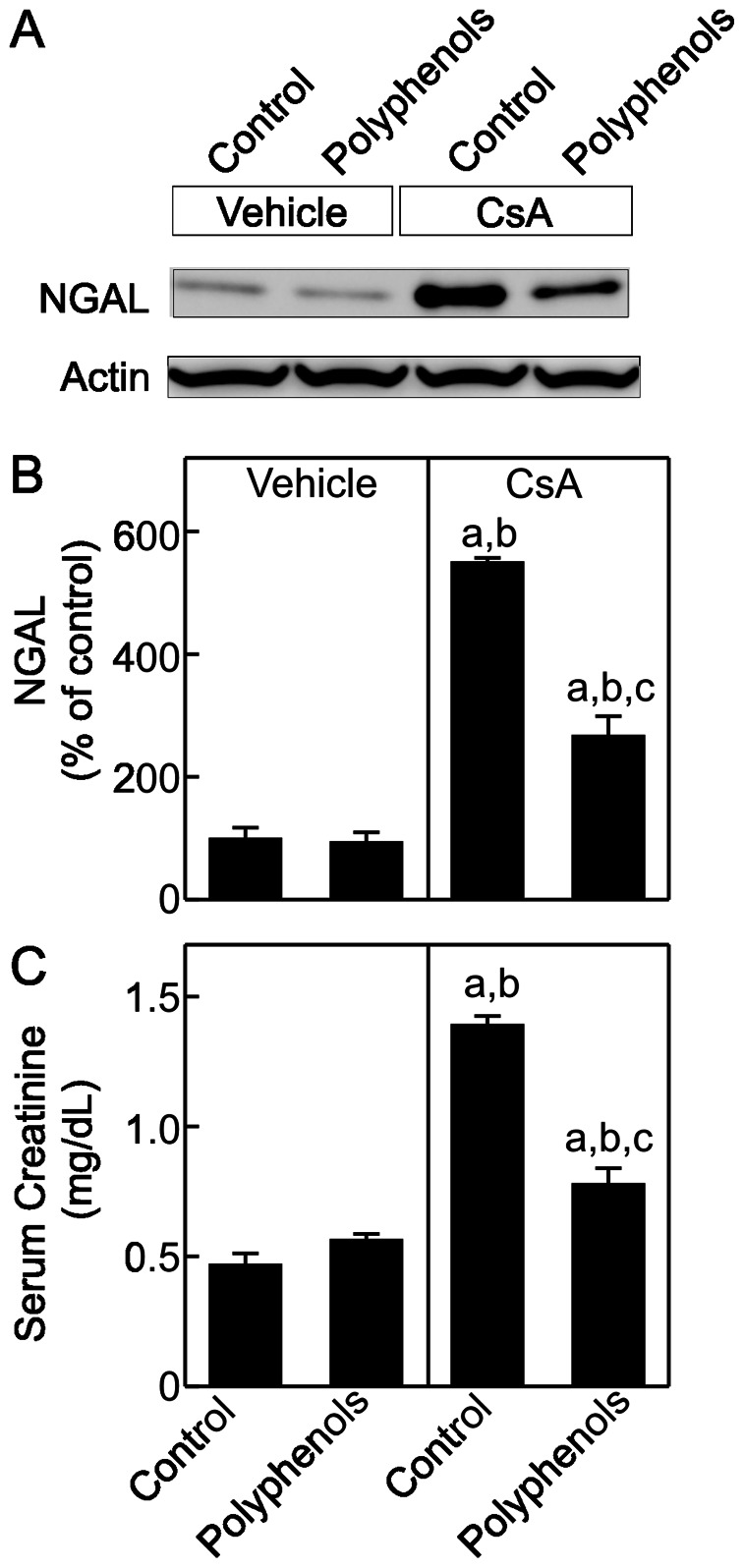
Polyphenols attenuate kidney injury and improve renal function after CsA treatment. Rats were fed semi-synthetic powdered diets containing 0% (**Control**) or 0.1% polyphenol extracts (**Polyphenols**) starting 3 days prior to CsA treatment (25 mg/kg, *i.g.* for 3 weeks). Neutrophil gelatinase-associated lipocalin (**NGAL**, 23 kDa) and actin in renal tissue were determined by immunoblotting. Representative images are shown in **A** and quantification by densitometry is shown in **B.** Serum creatinine was determined colorimetrically (**C**). a, p<0.05 *vs.* control diet+vehicle; b, p<0.05 *vs.* polyphenol-containing diet+vehicle; c, p<0.05 *vs.* control diet+CsA (n = 4 per group).

Neutrophil gelatinase-associated lipocalin (NGAL), a sensitive marker of acute kidney injury and a potential indicator of chronic kidney disease progression [61]–[64], was barely detectable in vehicle-treated rats with or without polyphenol treatment. NGAL increased by 5.5-fold after CsA treatment (Fig. 10 A and B). This effect was blunted by polyphenols.

Serum creatinine was 0.47 mg/dL in vehicle-treated rats and was not altered by polyphenols alone (Fig. 10C). After CsA treatment, serum creatinine increased to 1.4 mg/dL and polyphenols decreased serum creatinine to 0.78 mg/dL in CsA-treated rats. Together, CsA-induced pathological changes in the kidney were consistent with tubulointerstitial injury and these effects were markedly attenuated by polyphenols.

## Discussion

### Suppression of MB by CsA Treatment

The calcineurin inhibitor CsA is the basis for many immunosuppressive protocols, but its adverse effects (i.e. severe nephrotoxicity) are a major barrier in long-term immunosuppressive therapy [4], [5], [7]. The mechanisms of CsA nephrotoxicity are not well understood. CsA at high concentrations inhibits respiration and damages proteins and lipids in isolated mitochondria [17], [65], [66]. After in vivo treatment CsA decreases ATP levels in the rat kidney [19], [21]. Decreased oxidative phosphorylation could cause cell damage and inhibit renal function. Another important side effect of CsA, neurotoxicity, is linked to decreased mitochondrial metabolism [20], [67].

Mitochondrial homeostasis is crucial for maintaining proper energy supply and function of tissues. The abundance of mitochondria in a cell is determined by biogenesis, fission/fusion, and mitophagy [56]. When increased tissue energy demand exceeds mitochondrial ATP-producing capacity (e.g. exercise) or replacement of damaged mitochondria is needed, MB is stimulated. Mitochondria cannot be made *de novo* but require synthesis of new organelle constituents and the integration of these components (i.e., proteins and lipids) into preexisting mitochondria. MB requires synthesis and import of nDNA-encoded OXPHOS proteins (e.g. AS-β and COX-IV) into mitochondria as well as expression of mtDNA-encoded OXPHOS proteins (e.g. ND3) [56], [68], [69]. Suppression of MB could sensitize a tissue to toxicants and diseases.

A previous study showed that low dose of CsA protected against doxorubicin-induced heart dysfunction but did not alter MB [70]. In contrast, we observed that after chronic CsA treatment, mtDNA copy number was decreased substantially (Fig. 1), which was accompanied by a marked reduction in nDNA- and mtDNA-encoded OXPHOS proteins and their associated mRNAs (Figs. 2–3). Taken together, these data are consistent with the conclusion that renal MB is suppressed after CsA treatment.

### CsA Treatment Decreases PGC-1α Expression and Activity

MB is tightly regulated by a signaling system connecting different pathways [49], [71]–[74]. The transcriptional coactivators (PGC-1α and β) and the PGC-1-related coactivator (PRC) modulate the expression of target genes encoding OXPHOS enzymes [58], [75], [76]. PGC-1α induces and coordinates expression of nuclear regulatory proteins (e.g. nuclear respiratory factor (NRF)-1 and NRF-2) that activate target genes encoding OXPHOS proteins, PGC-1α itself, and Tfam. Tfam is a transcription factor that acts on the promoters within the non-coding (D-loop) region of mtDNA and regulates the replication and transcription of the mitochondrial genome [59], [77]. Over-expression of PGC-1α leads to mitochondrial proliferation in the heart, adipocytes, myoblasts and renal proximal tubular cells [47], [50], [76], [78], [79]. AMP-activated kinase (AMPK), sirtuin 1 (SIRT1), nitric oxide and cGMP, and other signaling kinases (e.g. Ca^2+^/calmodulin-stimulated protein kinase (CaMK), p38 MAPK and protein kinase C) regulate PGC-1α expression and/or activity [80]–[84].

Studies showed that calcineurin activation increases PGC-1α gene transcription [22]. Also, over-expression of constitutively active calcineurin in mouse skeletal muscle or cardiac myocytes leads to increased expression of PGC-1α [23], [24]. A coordinate increase of PGC-1α and its downstream transcription factors as well as gene expression of mitochondrial proteins were observed in association with calcineurin activation in human muscle after long term exercise [85]. PGC-1α and calcineurin activation also play an important role in MB in both healthy and diseased human skeletal muscles [85], [86]. Calcineurin stimulates members of the myocyte enhancer factor 2 (MEF2) family of transcription factors which bind to and activate the PGC-1α promoter and enhance a positive feedback loop between PGC-1α and MEF2 in muscle [22], [87]. In contrast, little is known concerning the effects of calcineurin inhibition on MB and mitochondrial homeostasis. In this study chronic exposure to CsA altered PGC-1α signaling in the kidney by decreasing PGC-1α protein and mRNA, and increasing PGC-1α acetylation (Figs. 4–5). PGC-1α also controls the expression of Tfam and Tfam decreased after CsA treatment (Fig. 6), consistent with decreased mtDNA copy number and suppressed expression of mtDNA-encoded ND3 (Figs. 1 and 3). In total, these data are consistent with PGC-1α depletion mediated suppression of MB. Because mitochondria are abundant in proximal tubular cells, decreases in mitochondrial OXPHOS proteins and pathological changes after CsA treatment were most overt in these cells (Figs. 2, 7 and 8).

### Green Tea Polyphenols Enhance MB after CsA Treatment

Stimulation of MB could counteract disease- or toxicant-induced mitochondrial suppression, enhance recovery of mitochondrial function, decrease tissue injury and promote tissue repair and regeneration. Some natural and synthetic molecules have been found to stimulate MB, such as small molecule SIRT1 activators, nitric oxide, isoflavones, β_2_-adrenergic receptor agonists, AMPK activators, cAMP and cGMP analogues, and 1-(2,5-dimethoxy-4-iodophenyl)-2-aminopropane [41], [46], [88]–[92]. Epicatechin improves MB and attenuates mitochondrial dysfunction in rodents and in patients with diabetes and chronic heart failure [42], [43]. Epicatechin also enhances fatigue resistance and oxidative capacity in aged mouse muscle [44]. Here we investigated the effect of green tea polyphenols, which was shown to protect against CsA nephrotoxicity [31], on MB. Polyphenols increased molecules regulating MB (PGC-1α, Tfam), mtDNA and OXPHOS proteins in control and CsA-treated rats, and these effects were associated with decreased kidney injury and improved renal function after CsA treatment (Figs. 7, 8, 9, 10). Therefore, in addition to their effects as antioxidants, green tea polyphenols may also protect and/or promote renal function by stimulating MB.

Interestingly, in addition to increasing PGC-1α mRNA and protein, polyphenols also increased PGC-1α activation as indicated by decreased acetylated PGC-1α (Fig. 5). This reduction in acetylated PGC-1α was not due to decreased PGC-1α protein since PGC-1α was equally loaded in the gels for immunoblotting after immunoprecipitation. A similar effect was observed in isoflavone-treated renal proximal tubular cells, isoflavones increased activity and protein content of SIRT1, a member of the histone deacetylase (HDAC) family [41]. Green tea polyphenol epigallocatechin gallate also increases HDAC activity and HDAC-2 expression in regulatory T cells [93]. Thus polyphenols may increase SIRT1 activity, decrease PGC-1α protein acetylation and increase PGC1α transcription.

A recent study showed that green tea polyphenols can bind to and stimulate a 67-kDa laminin receptor, leading to activation of NADPH oxidase and generation of reactive oxygen species in PC12 cells subjected to oxygen-glucose deprivation [94]. Red wine polyphenols at a low concentration but not at a high concentration stimulated MB and angiogenesis, and this effect depended on the estrogen receptor-α activation [95]. However, it is unclear whether estrogen receptor-α activation by red wine polyphenols is a direct or indirect effect. As discussed above, isoflavones have been shown to increase SIRT1 activity and protein content and may induced MB through this mechanism. In the present study, polyphenols increased MB in tubular cells which originally have abundant mitochondria but did not increase MB in glomeruli which have lower levels of mitochondria, and this stimulation of MB depended on the PGC-1α signaling pathway. Whether polyphenols act directly or indirectly to increase MB and whether this stimulation of MB requires binding of polyphenols to a specific receptor remain to be investigated.

The green tea extract contains several polyphenols. The major polyphenol in the extract was epigallocatechin gallate (∼50%). Our previous studies showed that epicatechin and epicatechin gallate had similar protective effects on liver ischemia/reperfusion injury and liver transplantation as green tea extracts containing multiple polyphenol components [29], [30]. In some other studies, the relative activities of the various polyphenolic components to inhibit oxidation and injury were variable [96]. It was also shown that a combination of epigallocatechin gallate, epicatechin gallate, epigallocatechin, and epicatechin in the molar ratio 5∶2:2∶1 provided optimal protective effects against lipid peroxidation [97]. Studies should be performed in the future to evaluate the efficacies of each polyphenolic component and various combinations of polyphenols on MB in cultured renal cells and in vivo.

### Conclusion

Taken together, MB is suppressed in the kidney after chronic CsA treatment, which may contribute to the development of CsA nephrotoxicity. Green tea polyphenols protect against CsA nephrotoxicity, at least in part, by enhancing MB.
